# Use of methotrexate in the management of recurrent Tolosa-Hunt syndrome: Two case reports

**DOI:** 10.1097/MD.0000000000019882

**Published:** 2020-04-24

**Authors:** Hyuk Sung Kwon, Tae Yoon Kim, Ji Hyun Kim, Jeong Hoon Bae, Seong-Ho Koh, Hojin Choi, Kyu-Yong Lee, Young Joo Lee, Young Seo Kim, Hyun Young Kim

**Affiliations:** a Department of Neurology; b Department of Radiology, Hanyang University Guri Hospital, Guri, Republic of Korea.

**Keywords:** methotrexate, prognosis, Tolosa-Hunt syndrome, treatment

## Abstract

**Rationale:**

Tolosa-Hunt syndrome (THS) is rare condition characterized by painful ophthalmoplegia that usually responds well to corticosteroid. About a half of THS patients experience recurrence within intervals of months to years from initial presentation. Recurrence is more common in younger patients, and can be ipsilateral, contralateral, or bilateral. Cyclosporine, azathioprine, methotrexate, mycophenolate mofetil, infliximab, and radiotherapy can be considered as second-line treatment. However, there is insufficient evidence for treatments preventing recurrence of THS.

**Patient concerns:**

We experienced two patients with THS that recurred twice while tapering or after ceasing corticosteroid administration.

**Diagnosis:**

Both patients were diagnosed as recurrent THS.

**Interventions:**

Methotrexate was treated with a combination of corticosteroid after THS recurred twice with corticosteroid therapy alone.

**Outcomes:**

After adding methotrexate to the steroid regimen, their symptoms were successfully regulated and ceased to recur

**Lessons:**

These cases add to the evidence for the use of methotrexate as a second-line therapeutic agent for those patients with recurrent THS attacks. Further studies are in need to prove the risk and benefits of second-line treatments in THS.

## 1 Introduction

Tolosa-Hunt syndrome (THS) is a rare condition characterized by unilateral periorbital or orbital pain associated with ophthalmoplegia. Although etiopathogenesis of THS is not elucidated, many pathological studies show granulomatous inflammation of the cavernous sinus, superior orbital fissure, or the orbit.^[1]^ THS usually responds well to corticosteroid treatment.^[2]^ However, recurrent attacks occur in approximately half of patients over an interval of months to years.^[3,4]^ But with no clear guidelines for preventing recurrences or treatment. We report 2 patients with THS who recurred 2 times and were successfully regulated with use of methotrexate.

## 2 Methods

Two patients who suffered from recurrent THS while tapering or after ceasing corticosteroid, and added methotrexate were included in this study. Informed consent was obtained from all individual participants included in the study. The diagnostic criteria for the diagnosis of THS was according to the 3rd edition of the International Classification of Headache Disorders as follow^[1]^;

A. Unilateral headache meeting criterion C.
B. Both of the following:
  (1) Granulomatous inflammation of the cavernous sinus, superior orbital fissure or orbit, demonstrated by magnetic resonance imaging (MRI) or biopsy.
  (2) Paresis of one or more of the third, fourth, and/or sixth ipsilateral cranial nerves.
C. Evidence of causality demonstrated by the following 2 elements:
  (3) Headache has preceded paresis of the third, fourth, and/or sixth nerves of ≤2 weeks, or developed with it.
  (4) Headaches is ipsilateral to the granulomatous inflammation
D. Not better accounted for by another ICHD-3 diagnosis.

## 3 Case reports

### 3.1 Case 1

**Figure 1:**
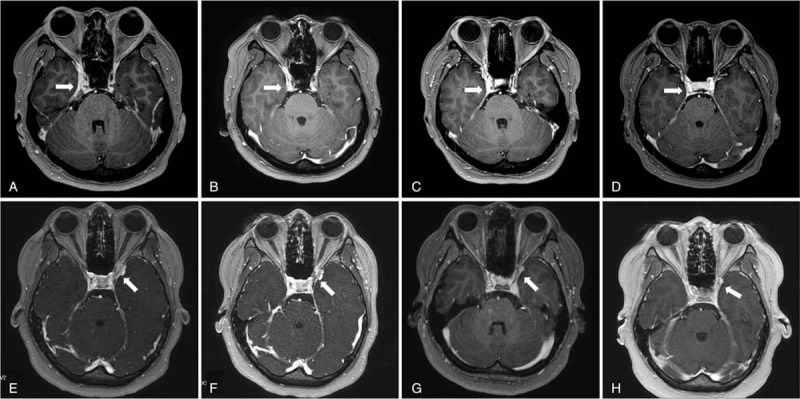
Serial gadolinium enhanced axial T1-weighted magnetic resonance images (MRI) of the patients (case 1: A–D, case 2: E–H). Enlargement and enhancement of right cavernous sinus and right superior orbital fissure was detected initially in 32-year-old woman (arrow, A). Two months later, lesion decreased markedly (B). Five months later, newly developed similar lesion was noted (C) and this lesion showed improvement 17 months later (D). MRI of 22-year-old woman showed small sized infiltrative mass at left superior orbital fissure (arrow, E). This lesion slightly decreased 2 months later (F). After second recurrence, more decreased lesion was seen (G). This lesion decreased more 8 months later, after adding methotrexate (H).

A 32-year-old previously healthy woman complained of headache in the right temporal area and diplopia. She showed medial- and up-gaze palsy of the right eye with orbital pain. The results of cerebrospinal fluid (CSF) study, serum angiotensin converting enzyme (ACE), neoplastic markers, and autoimmune antibodies were normal. Brain MRI showed enlargement and enhancement of the right cavernous sinus and right superior orbital fissure, suggesting an inflammatory condition such as THS (Fig. 1A). The patient was treated with oral prednisolone (1 mg/kg) and the dose was tapered over a period of 2 months. Follow-up MRI showed improvement of the previous lesion (Fig. 1B). Her symptoms recurred when prednisolone was tapered to 20 mg. Therefore, high dose prednisolone (1 mg/kg) was restarted and her symptoms improved again. There was a second recurrence 4 months later while tapering the steroid dose, with a newly developed lesion at the right cavernous sinus detected on MRI (Fig. 1C). The patient was treated with prednisolone 1 mg/kg for 7 days and tapered over a period of 5 months. Methotrexate was added at a dose of 7.5 mg weekly 2 months later and increased to 12.5 mg per week 4 months later. Both prednisolone (5 mg) and methotrexate were sustained for a total of 12 months and then stopped. Follow-up brain MRI showed resolution of the previous lesion (Fig. 1D). Clinical remission was achieved and has lasted for 3 years, until the time of this writing.

### 3.2 Case 2

A 22-year-old previously healthy woman complained of localized headache and diplopia. Vertical gaze palsy, ptosis, and mydriasis of the left eye were noted upon examination. Her results for CSF, serum ACE, neoplastic markers, and autoimmune antibodies were normal. Brain MRI showed an inflammatory lesion at the left superior orbital fissure area (Fig. 1E). She was treated with methylprednisolone (1 g/d for 5 days) followed by prednisolone (1 mg/kg), with the dose tapered over 2 months resulting in complete remission of clinical symptoms. However, she presented with diplopia and headache a few days after ceasing steroid treatment. Prednisolone (1 mg/kg) was restarted, and the dose was tapered over a period of 3 months. Her clinical symptoms improved again, and MRI revealed slight improvement (Fig. 1F). A second recurrence occurred after cessation of steroid treatment without newly developed lesions on MRI (Fig. 1G). The patient was treated with prednisolone 1 mg/kg and tapered over a period of 4 months. We added methotrexate 7.5 mg per a week a month later. Methotrexate treatment was sustained for 6 months at the same dosage, and steroid treatment was stopped 2 months before the cessation of methotrexate. Clinical remission was achieved and has lasted for 2 years until the time of this writing. Her final MRI revealed that the lesion had decreased further in size (Fig. 1H).

## 4 Discussion

We report 2 cases with successful clinical remission of recurrent THS after treatment with steroids followed by methotrexate. Both cases recurred twice while tapering or after ceasing steroid treatment. After adding methotrexate, their symptoms ceased to recur.

Spontaneous remission of symptoms may occur in THS. Treatment with corticosteroid usually relieves pain within 3 days and paresis within 1 week.^[3]^ Although THS has relatively favor prognosis, 30% to 50% of cases may recur. Recurrence is more common in younger patients, can occur from months to years after the initial attack, and can be ipsilateral, contralateral, or bilateral.^[3,4]^

For patients showing refractory to steroid or that require prolonged high-dose steroids, steroid-sparing agents such as cyclosporine, azathioprine, methotrexate, mycophenolate mofetil, or infliximab are considered as second-line treatments.^[5–8]^ Radiotherapy has also been used as a second-line therapy.^[6]^ However, there is insufficient evidence for the efficacy of these second-line treatments.

In a previous report, 4 of 20 patients with recurrent THS developed chronic pain and were treated satisfactorily by adding azathioprine.^[5]^ Another case report described the addition of azathioprine to steroid treatment in a patient with recurrent THS, but the patient's pain was not relieved completely and a new recurrence was noted. Therefore, radiotherapy was started and resulted in reduction of the lesion on brain MRI alongside improvement of symptoms that lasted for >3 years after treatment.^[6]^ Another study evaluated the effects of methotrexate (7.5 mg per week orally and increased up to 15 mg per week) in patients with non-infectious orbital inflammatory disease who failed to respond to systemic corticosteroids and/or orbital irradiation.^[7]^ Among 14 patients, 9 patients, including 1 THS patient, derived clinical benefit from methotrexate, 1 patient showed no response, and 2 patients ceased methotrexate due to side effects. Mycophenolate mofetil was used successfully in 5 patients with idiopathic orbital inflammation.^[8]^ Infliximab resulted in favorable response in a patient with THS who was refractory to corticosteroid. Infliximab was infused in a patient with relapsing THS, whose symptoms resolved without recurrence.^[2]^ In a recent retrospective study of THS, adding second-line treatment was suggested to lower the rate of recurrence.^[9]^

Methotrexate is a standard treatment for rheumatoid arthritis. Although it is widely used in clinical practice, the mechanism of action is poorly understood. It is an inhibitor of dihydrofolate reductase and thereby works as a folic acid antagonist. By inhibiting the synthesis of lymphotoxin, the chemotaxis of monocytes and the production of superoxide may contribute to its anti-inflammatory properties. Accumulation of polyglutamated metabolites that persist in tissues is involved in the adenosine-mediated anti-inflammatory effect and explains the prolonged anti-inflammatory effect of methotrexate.^[10]^ This prolonged anti-inflammatory effect might contribute to the regulation of recurrent THS. Our cases showed successful clinical remission of recurrent THS with use of methotrexate. No adverse effects were noted.

Our patients experienced successful clinical remission of recurrent THS with the use of methotrexate. No adverse effects were noted. These cases add to the existing evidence for the use of methotrexate as a second-line therapeutic agent for patients experiencing recurrent THS attacks. Further studies are needed to confirm the risks and benefits of second-line treatments in THS.

## Author contributions

**Conceptualization:** Hyuk Sung Kwon, Hyun Young Kim.

**Data curation:** Ji Hyun Kim, Jeong Hoon Bae.

**Investigation:** Hyuk Sung Kwon, Tae Yoon Kim, Ji Hyun Kim, Jeong Hoon Bae, Hojin Choi, Kyu-Yong Lee, Young Joo Lee, Hyun Young Kim.

**Supervision:** Seong-Ho Koh, Hojin Choi, Kyu-Yong Lee, Young Joo Lee, Young Seo Kim, Hyun Young Kim.

**Visualization:** Hyuk Sung Kwon, Tae Yoon Kim.

**Writing – original draft:** Hyuk Sung Kwon, Tae Yoon Kim.

**Writing – review & editing:** Seong-Ho Koh, Young Seo Kim, Hyun Young Kim.

Hyuk Sung Kwon: 0000-0002-2005-0983.

Tae Yoon Kim: 0000-0001-8392-1822.

Ji-Hyun Kim: 0000-0003-3299-7928.

Jeong Hoon Bae: 0000-0002-9843-8902.

Seong-Ho Koh: 0000-0001-5419-5761.

Hojin Choi: 0000-0002-9637-4423.

Kyu-Yong Lee: 0000-0001-8855-7513.

Young Joo Lee: 0000-0002-7531-9011.

Young Seo Kim: 0000-0002-7050-3426.

Hyun Young Kim: 0000-0003-2105-1547.
